# Validations of apomorphine-induced BOLD activation correlations in hemiparkinsonian rhesus macaques

**DOI:** 10.1016/j.nicl.2019.101724

**Published:** 2019-02-18

**Authors:** J.E. Quintero, Yi Ai, A.H. Andersen, P. Hardy, R. Grondin, Z. Guduru, D.M. Gash, G.A. Gerhardt, Z. Zhang

**Affiliations:** a Department of Neuroscience, University of Kentucky Chandler Medical Center, Lexington, KY 40536-0098, USA; b Magnetic Resonance Imaging and Spectroscopy Center, University of Kentucky Chandler Medical Center, Lexington, KY 40536-0098, USA; c Department of Neurology, University of Kentucky Chandler Medical Center, Lexington, KY 40536-0098, USA

**Keywords:** fMRI, Pharmacological MRI, Neurochemistry, Parkinson's disease

## Abstract

Identification of Parkinson's disease at the earliest possible stage of the disease may provide the best opportunity for the use of disease modifying treatments. However, diagnosing the disease during the pre-symptomatic period remains an unmet goal. To that end, we used pharmacological MRI (phMRI) to assess the function of the cortico-basal ganglia circuit in a non-human primate model of dopamine deficiency to determine the possible relationships between phMRI signals with behavioral, neurochemical, and histological measurements. Animals with unilateral treatments with the neurotoxin, 1-methyl-4-phenyl-1,2,3,6-tetrahydropyridine (MPTP), that expressed stable, long-term hemiparkinsonism were challenged with the dopaminergic receptor agonist, apomorphine, and structure-specific phMRI blood oxygen level-dependent (BOLD) activation responses were measured. Behavioral, histopathological, and neurochemical measurements were obtained and correlated with phMRI activation of structures of the cortico-basal ganglia system. Greater phMRI activations in the basal ganglia and cortex were associated with slower movement speed, decreased daytime activity, or more pronounced parkinsonian features. Animals showed decreased stimulus-evoked dopamine release in the putamen and substantia nigra pars compacta and lower basal glutamate levels in the motor cortex on the MPTP-lesioned hemisphere compared to the contralateral hemisphere. The altered neurochemistry was significantly correlated with phMRI signals in the motor cortex and putamen. Finally, greater phMRI activations in the caudate nucleus correlated with fewer tyrosine hydroxylase-positive (TH^+^) nigral cells and decreased TH^+^ fiber density in the putamen. These results reveal the correlation of phMRI signals with the severity of the motor deficits and pathophysiological changes in the cortico-basal ganglia circuit.

## Highlights

• Apomorphine in hemiparkinsonian animals can evoke changes in functional MRI signals.
• Cortico-basal ganglia activation correlates to behavior, neurochemistry, histology
• Pharmacological MRI has potential to be biomarker for Parkinson's disease.

## 1 Introduction

Identification of Parkinson's disease (PD) at the earliest possible stage of the disease may provide the best opportunity for the use of disease modifying treatments. However, correctly diagnosing the disease during the pre-symptomatic period remains difficult. This is, in part, because of the absence of predictive, non-invasive, in vivo tests for PD and because PD symptoms resemble symptoms of other neurological conditions. Moreover, the prevalence of both parkinsonian symptoms and PD, per se, increases with age. The ideal diagnostic test would confirm the presence of prodromal PD and provide some information about the lead time to motoric symptoms and the rate of progression.

Pharmacological MRI (phMRI) has been increasingly used as a neuroimaging technique to investigate region-specific brain activity associated with the administration of CNS-active drugs (Breiter et al., 1997; Grasby et al., 1993; Lahti et al., 1995). As an imaging modality, phMRI has a number of advantages related to molecular, structural, and functional neuroimaging that are rapidly expanding the neurobiological understanding of the complexities of PD. phMRI holds the promise of providing early indications of the pharmacodynamic activity of novel CNS-targeted compounds in a safe, non-invasive manner through blood oxygenation level dependent (BOLD) imaging in experimental animals (Andersen et al., 2015; Zhang et al., 2018), and in humans (Barber et al., 2017; Nemoto et al., 2017). As a result, significant effort has been devoted to implementing phMRI as a translational research tool to develop novel CNS compounds (Bifone and Gozzi, 2012; Wise and Tracey, 2006) and to study neurological disorders (Andersen et al., 2015; Seibyl et al., 2012; Weiller et al., 2006). Drug-induced pharmacodynamic responses detected from alterations in BOLD signals or relative cerebral blood volume/flow (rCBV/rCBF) can serve as imaging biomarkers to delineate the central effect of drug actions (Bhattacharyya et al., 2017; Ghariq et al., 2014; Jann et al., 2015; Tak et al., 2015). In addition, phMRI has been used to study several key neurotransmitter systems. Collectively, these studies have highlighted the value of phMRI to elucidate neurobiological mechanisms of drug actions to gain a better understanding of PD (Seibyl et al., 2012).

However, the clinical use of phMRI techniques as a tool for the early diagnosis and clinical progression of PD is still lagging compared with other modalities like positron emission tomography (PET) or single photon emission computed tomography (SPECT), possibly because the underlying neurophysiological mechanism(s) of phMRI signals are not fully understood. Consequently, determining the relationships between phMRI BOLD-activation and disease-related changes in neurobiology and neurophysiology may lead to a more integrated and comprehensive understanding of PD (Weingarten et al., 2015). Pharmacological neuroimaging as it relates to dopamine terminal degeneration could serve important and unique roles in both earlier and later stages of the disease. In the early stages, neuroimaging might potentially allow diagnosis in the prodromal state. In the later stages, it might help with elucidating pathology relevant to new drug development.

In the present study, the nonselective dopamine D_1_/D_2_ receptor agonist apomorphine (APO; currently used as an FDA-approved PD treatment) was used to stimulate the dopaminergic systems in animals with stable, long-term MPTP-induced parkinsonism. The phMRI responses in the cortico-basal-ganglia-cortical circuit were directly correlated with a variety of in vivo measurements including behavioral responses, in vivo microdialysis measurements of dopamine (DA) function, and electrochemical recordings of resting glutamate levels. Additional comparisons were made with postmortem data including dopaminergic neuron counts in the substantia nigra (SN) pars compacta (SNc) and fiber density in the striatum. These experiments were all designed to test the hypothesis that phMRI signals (BOLD responses) correlate with the severity of the motor deficits and pathophysiological changes in the nigrostriatal system and could be used as an indicator of biological processes that change in response to dysfunction within the cortico-basal-ganglia-cortical circuit.

## 2 Methods and materials

### 2.1 Animals

All procedures used in the study were approved by the University of Kentucky Institutional Animal Care and Use Committee and followed NIH and USDA guidelines. Six, late middle-aged (18 to 22 years old), female rhesus monkeys (*Macaca mulatta*) expressing long-term hemiparkinsonism were used in the study. All animals were originally obtained from a commercial supplier (Covance, Alice, TX) and were housed in a temperature-controlled room and maintained on a 12-h light and 12-h dark cycle. Throughout the entire study, water was available ad libitum and standard non-human primate biscuits were supplemented daily with fresh fruits and vegetables.

Data were obtained within an 18-month period, approximately 3.5 years after the animals had been rendered hemiparkinsonian by a unilateral administration of 0.12 mg/kg MPTP (1-methyl-4-phenyl-1,2,3,6-tetrahydropyridine) via the right carotid artery. After MPTP administration, all animals developed mild to moderate parkinsonian features including bradykinesia, rigidity of upper and lower limbs on the affected side, stooped posture, and mild postural instability. Some data shown here were previously reported and are recounted here to describe the behavioral, histological, and neurochemical deficits of these animals and provide the basis for correlative analyses.

### 2.2 Behavioral evaluation

All animals were extensively monitored and evaluated by non-biased, objective, and non-invasive methods including an automated video-tracking system (EthoVision Pro, Noldus Technologies, Asheville, NC) and Actical accelerometers (MiniMitter, Bend, OR). *Automated Movement Tracking.* The detailed procedures have been described elsewhere (Ding et al., 2008; Luan et al., 2008; Walton et al., 2006; Xin et al., 2008). Briefly, video recording procedures were conducted in specially-designed cages located in a room adjacent to the housing quarters. The tracking method relies on calibrating the software to the dimensions of the cage, so that the position of the animal can be determined in the cage based on (*x,y,z)* coordinates. Behavioral values, including distance traveled (cm) and movement velocity (cm/s) were measured over the course of one hour. The same coded video records were also used by trained observers to blindly evaluate parkinsonian features using our nonhuman primate parkinsonian rating scale (Ding et al., 2008; Ovadia et al., 1995). *Actical accelerometers.* Changes in overall home-cage activity level were continuously monitored for each monkey using activity monitors worn on collars. The monitors were programmed to measure and store the activity counts at a one-minute sample interval. Daily activity counts were recorded from 06 h00 (lights ON) to 18 h00 (lights OFF) and nighttime levels were measured from 18 h00 to 06 h00.

### 2.3 Pharmacological MRI (phMRI)

Based on previously published procedures (Zhang et al., 2018), all images were acquired on a Siemens 3 T Trio clinical MRI system using a dedicated receive-only coil for reception, which was designed and developed by our group. Animals were maintained under general anesthesia (~1% isoflurane in oxygen) during image acquisitions. The BOLD-effect weighted MR images used to measure the phMRI response were acquired in an anatomically coronal plane. The image planes of the acquisition were arranged to cover the motor cortex and the basal ganglia. A segmented gradient-echo EPI sequence with TE = 28 ms and a turbo factor of 7 was used to reduce echo train length and minimize susceptibility-related artifacts. The EPI sequence acquisition parameters are FOV = 112 × 98 mm and image matrix 64 × 56 for an in-plane resolution of 1.75 mm. A total of 15 contiguous slices, each 2 mm-thick, were acquired at a rate of 15 s per EPI volume. The overall scan duration was 80 min with 128 volumes acquired prior to APO administration (0.1 mg/kg injection S.C.) as a baseline and 192 volumes acquired after APO to track the response. APO was prepared daily in 0.9% sterile saline 2 h before the scan. Images were motion corrected and spatially smoothed using a Gaussian kernel of width 3.5 mm. phMRI response was calculated as the fractional signal change in % of the average of the post-APO image data relative to the pre-APO baseline. A co-registered high-resolution (0.67 × 0.67 × 1 mm) T1-weighted anatomical MRI scan was acquired in each session for spatial localization of the activation response.

### 2.4 In vivo microdialysis of dopamine overflow

Measurement of both potassium- and d-amphetamine-induced DA release was collected following previously published procedures from each animal, after the PD features were fully developed and stable (Cass et al., 2007; Gerhardt et al., 2002; Grondin et al., 2003). Briefly, custom-made CMA 11 dialysis probes with a membrane length of 3.0 mm and diameter of 0.24 mm (CMA Microdialysis, North Chelmsford, MA) were slowly lowered under sterile conditions into the putamen or SNc using MRI-derived stereotactic coordinates for each anesthetized animal. The probes were perfused at a flow rate of 1.2 μl/min with artificial CSF, and dialysate fractions were collected at 30-min intervals. After a 1-h equilibration period, two baseline fractions were collected and then overflow of DA was locally stimulated by reverse microdialysis by switching to a perfusate solution containing elevated potassium (100 mM) for a single 30-min fraction, then switching back to the original perfusate for three additional fractions. A d-amphetamine (250 μM) stimulation was then included in the perfusate for one 30-min fraction followed by three final fractions with normal artificial CSF. Dialysate samples were assayed for DA after collection using HPLC with electrochemical detection (Cass et al., 2003). Dialysis probe placement was confirmed in each animal by structural MRI within 48–72 h post-surgery.

Basal levels of DA were defined as the average value of the two fractions preceding stimulation by excess potassium. All probes were calibrated in vitro prior to use to determine acceptable probes (recovery of DA at least 15%). However, values were not corrected for in vitro recoveries, as uncorrected values may be better correlated to true values (Glick et al., 1994). Dialysis data were expressed as nM concentration of DA or metabolite in the dialysate and, for evoked overflow, as the total amount of DA in the dialysate above baseline after stimulation with potassium or d-amphetamine.

## 3 Electrochemical recordings of basal levels of glutamate

Based on previously published procedures (Fan et al., 2014; Quintero et al., 2007), the resting levels of glutamate were measured, with biosensors, bilaterally in the primary motor cortex covering the trunk and upper body regions (Fig. 4a) on the same day as necropsy. Under general anesthesia (~1% isoflurane in oxygen), the skull directly over the primary motor cortex was removed bilaterally to allow access to the biosensors and a reference electrode (a Ag/AgCl electrode, Model RE-5B, Bioanalytical Systems, West Lafayette, IN) was inserted into a remote pocket (5 mm × 50 mm) in the area above the occipital lobes so as to not interfere with the biosensors. Five different points (2–3 mm apart) from medial to lateral primary motor cortex were used on each of the left and right sides of the cortex as illustrated in Fig. 4a. Resting glutamate values for the right (MPTP-lesioned) and left cortices were generated from an average of the last 30 s of the equilibration period.

### 3.1 Post-mortem morphology

Immediately after the glutamate recordings, all animals were deeply anesthetized with pentobarbital (20–25 mg/kg, IV) and euthanized via transcardial perfusion with 0.9% saline based on previously published procedures (Ding et al., 2008; Gash et al., 1996). The brains were immediately removed, immersion-fixed in 4% paraformaldehyde solution in 0.1 M phosphate buffer (pH 7.4) overnight and cryoprotected in 30% buffered sucrose until they sank. Then, 40 μm-thick sections were cut on a sliding microtome through the SN. Sections were processed for tyrosine hydroxylase (TH, monoclonal antibody, 1:1000; Chemicon International, Temecula, CA, USA) for assessment of TH-positive (TH^+^) neuronal loss in the midbrain and loss of TH^+^ fibers in the striatum (procedures described elsewhere, Ding et al., 2008; Gash et al., 1996; Grondin et al., 2002). The number of TH^+^ midbrain dopaminergic neurons was estimated bilaterally using an optical fractionator method for unbiased stereological cell counting. In addition, TH^+^ fiber density was measured as percentage of area with TH^+^ immunoreactive staining in both sides of the caudate nucleus and putamen of each hemisphere and in each animal.

### 3.2 Statistical analysis

Statistical comparisons between the right (MPTP-lesioned) and left hemispheres were treated as un-paired measurements as MPTP treatment profoundly affects the structure and physiology of the effected hemisphere (Ding et al., 2008; Hamadjida et al., 2018). The phMRI data used in the linear regression and correlation analyses were from the hemisphere (right) on the ipsilateral side of MPTP administration. All linear regressions were first order, and two-tailed Pearson correlations were used to identify relationships among behavioral, phMRI, or resting glutamate features. Glutamate resting levels were analyzed from time-series recordings in individual animals using custom MatLab®-based analysis software, averaged from the multiple points in each animal, and compared among animals at the group level using a Student's *t*-test. The in vivo microdialysis results were initially analyzed using an ANOVA followed by Newman–Keuls post hoc comparisons for the DA response at individual time points. All statistical analyses were conducted using Prism 6 or 7 GraphPad Software (San Diego, CA). Differences of *p* < 0.05 were considered significant.

## 4 Results

### 4.1 Dynamic phMRI response to APO

**Fig. 1:**
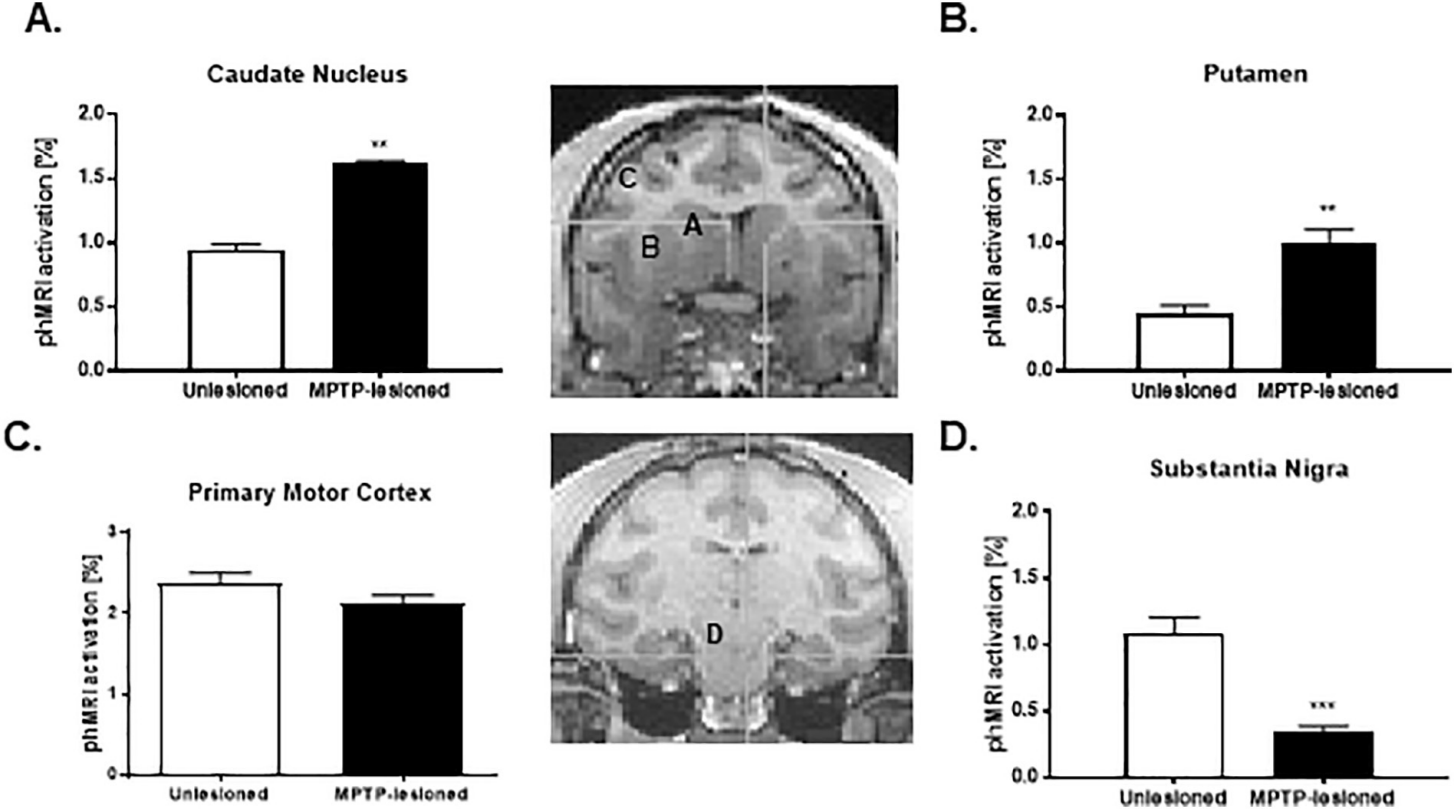
phMRI activation in different structures between the MPTP-lesioned and unlesioned hemispheres. (A) caudate nucleus, (B) putamen, (C) primary motor cortex, and (D) substantia nigra (SN). *p < 0.05, ** *p* < 0.01.

APO-evoked activations were observed in the caudate nucleus (Unlesioned: 0.95 ± 0.04% phMRI activation vs. MPTP-lesioned: 1.62 ± 0.02% phMRI activation; *P* < 0.01, Fig. 1A) and putamen (Unlesioned: 0.47 ± 0.37% phMRI activation vs. MPTP-lesioned: 1.11 ± 0.36% phMRI activation; *P* < 0.01, Fig. 1B). BOLD activation induced by the APO challenge in the primary motor cortex was lower but not significantly different (Unlesioned: 2.38 ± 0.13% phMRI activation vs. MPTP-lesioned: 2.12 ± 0.10% phMRI activation; *P* = 0.1; Fig. 1C). The second novel observation was the reduction of BOLD activation induced by the APO challenge in the SN (Unlesioned: 1.09 ± 0.12% phMRI activation vs. MPTP-lesioned: 0.35 ± 0.04% phMRI activation; *P* < 0.01; Fig. 1D). The result in the SN differs from previous findings in short-term PD monkeys in which activation was observed in the SN with lower doses of APO (Zhang et al., 2006).

### 4.2 Correlations with phMRI

#### 4.2.1 Behavior

**Fig. 2:**
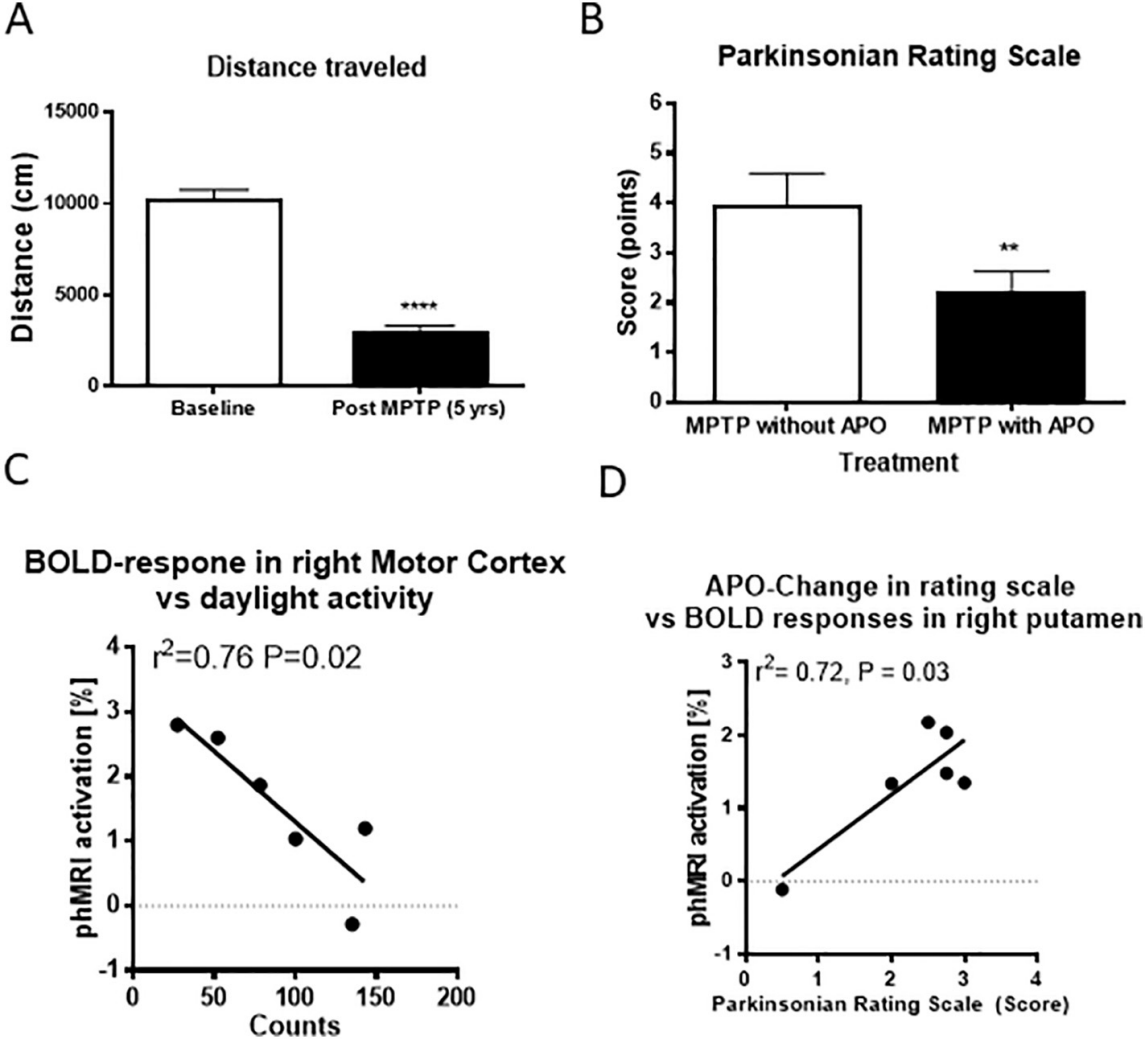
(A) Distance traveled between baseline and after MPTP lesion showed a significant (*p* < 0.001) decrease in distance traveled after MPTP lesion. (B) MPTP-treated animals showed characteristics associated with dopamine deficiency as measured with a parkinsonian rating scale. Long-term, stable parkinsonian features improved with apomorphine treatment; **P < 0.01. (C) Home cage activity was inversely correlated with phMRI signals in the right motor cortex. (D) Apomorphine-induced changes in the parkinsonian rating scale was positively correlated with BOLD activation in the right (MPTP-treated side) putamen.

All animals exhibited stable motor deficits as judged by distance traveled (Fig. 2A, Baseline: 10284 ± 503 cm vs. Post MPTP: 3060 ± 288 cm; *P* < 0.0001) and movement speed (Baseline: 2.9 ± 0.1 cm/s vs. Post MPTP: 1.5 ± 0.2 cm/s; *P* = 0.0002). In addition, all animals showed a positive response to APO (Fig. 2B). The total PD score on the nonhuman primate parkinsonian scale improved by over 40% post APO administration (Fig. 2B, MPTP without APO: 4.0 ± 0.6 points vs. MPTP with APO: 2.3 ± 0.4 points; *P* = 0.0075). phMRI responses in the putamen were negatively correlated with the distance traveled (*r*^*2*^ = 0.68, *P* = 0.042) and the movement speed (*r*^*2*^ = 0.69, *P* = 0.039). Similar relationships were also identified between phMRI responses in the motor cortex and the daytime home-cage activity (*r*^*2*^ = 0.76, *P* = 0.02, Fig. 2C). APO-induced behavioral changes in PD features were significantly correlated with APO-induced phMRI responses in the putamen (*r*^*2*^ = 0.72, *P* = 0.03, Fig. 2D), premotor cortex (*r*^*2*^ = 0.80, *P* = 0.015), and cingulate gyrus (*r*^*2*^ = 0.78, *P* = 0.018).

#### 4.2.2 Histopathology

**Fig. 3:**
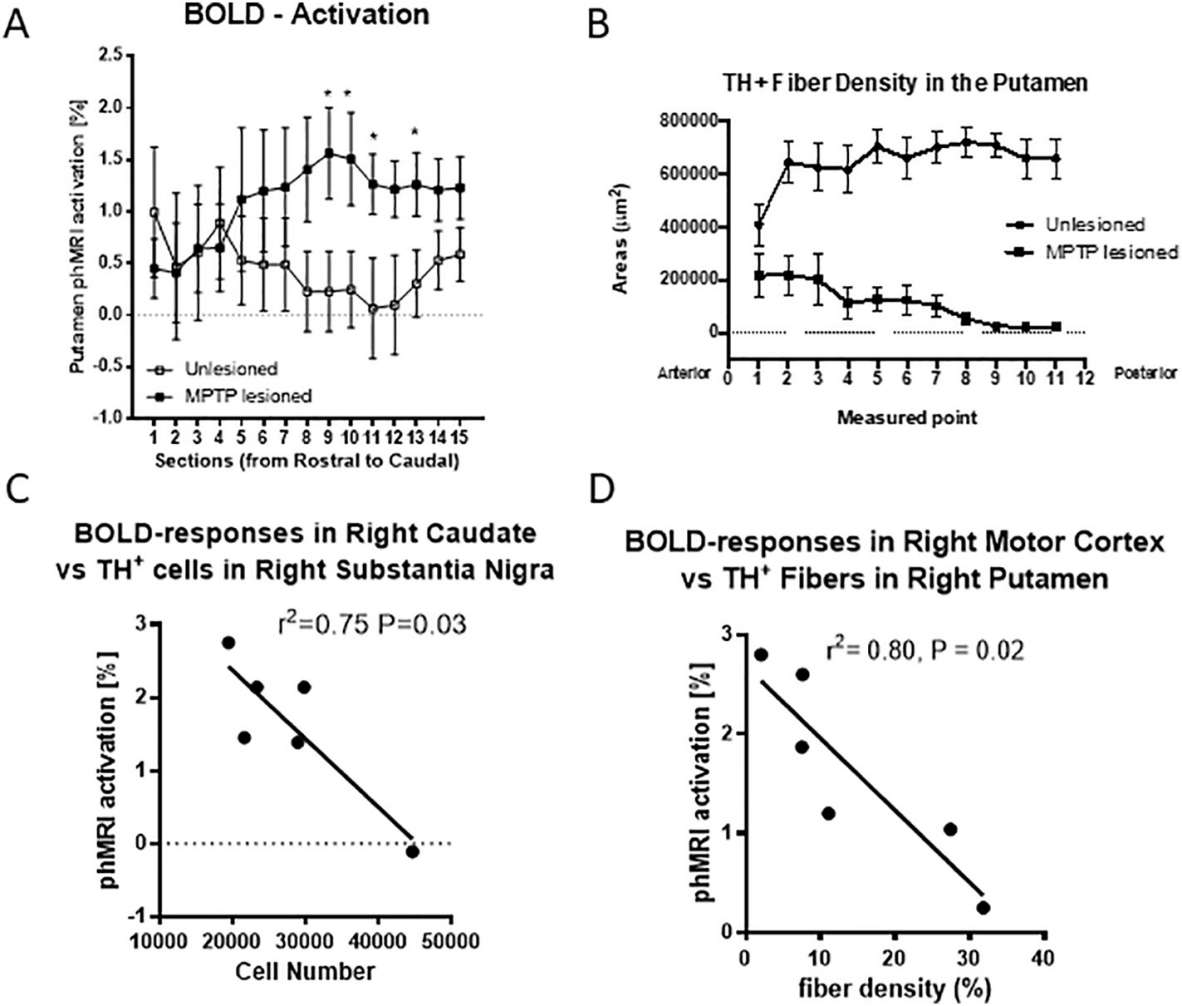
Areas of phMRI activation in the motor cortex and basal ganglia in (A) after a 20 min APO challenge. In the MPTP-lesioned side (), the putamen shows areas of higher APO-induced BOLD activation along the rostral-caudal axis than in the unlesioned side (). Sections were 3 mm in thickness through the putamen. *p < 0.05. (B) TH^+^ fiber density distribution along the anterior-posterior plane of the putamen in the MPTP-lesioned and the unlesioned hemispheres. (C) BOLD-responses in the right (MPTP-lesioned side) caudate were negatively correlated with the number of TH^+^ cells in the right substantia nigra. (D) BOLD-responses in the right motor cortex were negatively correlated with TH^+^ fibers in the right putamen.

A novel observation was the heterogeneous responses to APO challenge in the putamen on the ipsilateral side of MPTP administration (Fig. 3A). The greatest responses to APO in the MPTP-lesioned side were observed in the more posterior sections (8–13) while the responses in the last 2 sections (14–15) were smaller. Unbiased stereological cell counts of TH^+^ dopamine neurons revealed >85% loss of TH^+^ cells in the MPTP-treated SN; and densitometry of TH^+^ fiber showed a significant reduction of TH^+^ fibers density in the MPTP-treated putamen. Only 16.9% of dopaminergic fibers remained compared with the contralateral side. The loss of TH^+^ terminals in the putamen was heterogeneous as shown by a greater loss in the posterior than anterior sections (Fig. 3B). The number of TH^+^ cells in the SNc was negatively correlated with the phMRI-responses in the caudate nucleus (*r*^*2*^ = 0.75, *P* = 0.026, Fig. 3C). In addition, TH^+^ fiber densities in the lesioned putamen (*r*^*2*^ = 0.80, *P* = 0.016, Fig. 3D) and caudate nucleus (*r*^*2*^ = 0.66, *P* = 0.048) were significantly correlated with phMRI-responses seen in the primary motor cortex.

#### 4.2.3 Neurochemistry

**Fig. 4:**
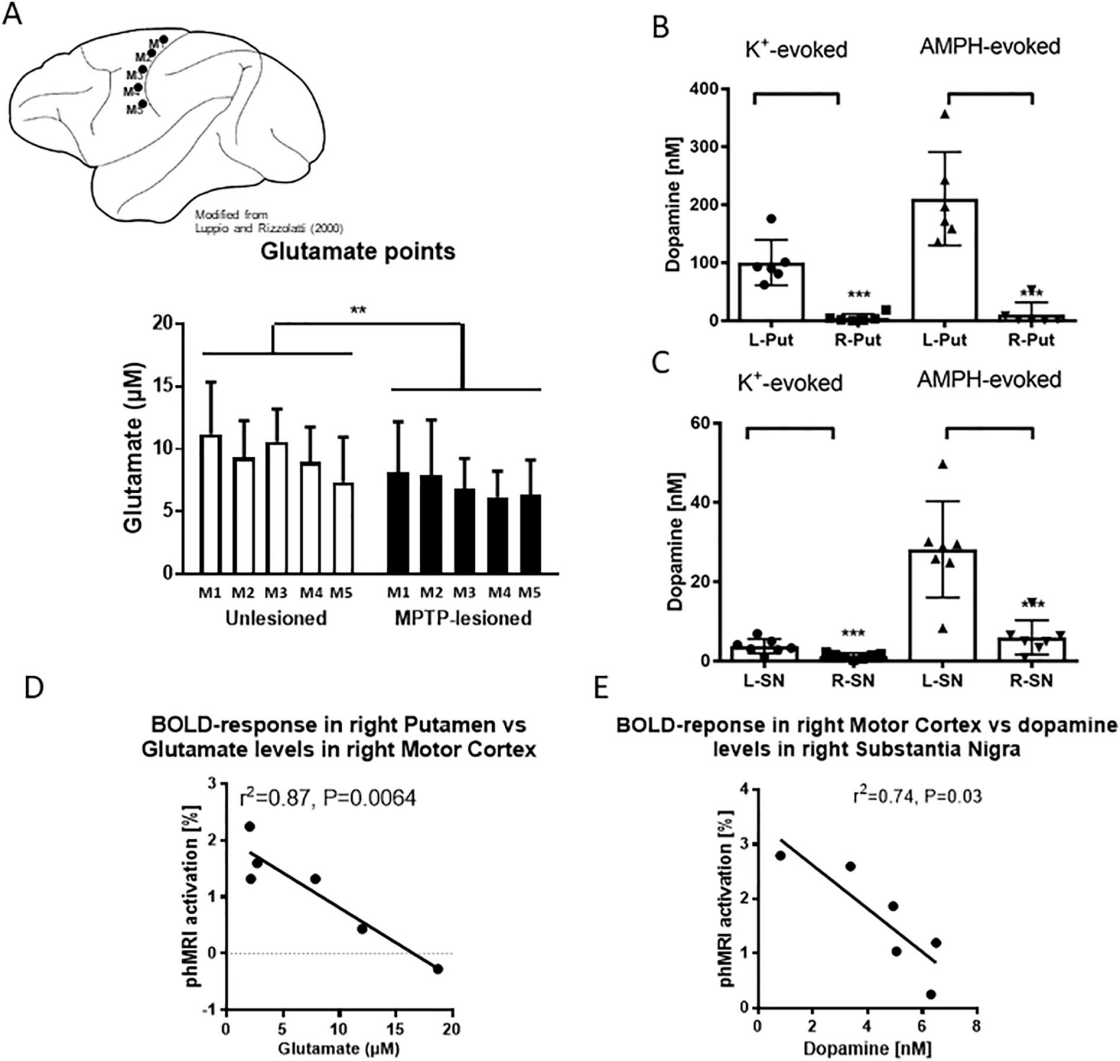
(A) Resting levels of glutamate were measured from 5 areas (2–3 mm apart) in the primary motor cortex from both the left and right hemispheres. Basal glutamate levels in the ipsilateral motor cortex were significantly lower than the contralateral motor cortex; *P* = 0.031. K+ (100 mM)- and amphetamine (250 μM)-evoked DA release was significantly attenuated in the ipsilateral (B) putamen (Put) and (C) SNc; ***P < 0.0001 – adapted from (Quintero et al., 2011). (D) Glutamate levels in the right (MTP-lesioned side) motor cortex were inversely correlated with phMRI signals in the right putamen. (E) BOLD-responses in the right motor cortex were negatively correlated with dopamine levels in the right subtantia nigra after amphetamine stimulation.

The average of individual resting basal glutamate (5 sample sites in each hemisphere) in the motor cortex was significantly lower in the MPTP-lesioned than the unlesioned hemisphere (Fig. 4A). Both potassium- and d-amphetamine-evoked overflow of DA in the putamen (Fig. 4B) and SNc (Fig. C) on the ipsilateral side of the lesion were lower than the contralateral side. Basal glutamate levels in the primary motor cortex were significantly and negatively correlated with phMRI activation in the putamen (*r*^*2*^ = 0.87, *P* = 0.0064, Fig. 4D) and in the premotor cortex (*r*^*2*^ = 0.83, *P* = 0.011). Both potassium- and d-amphetamine-evoked overflow of DA in the putamen (each measured for a single time point, 30 min after stimulus administration) had significant correlations with phMRI responses in the putamen (data not shown). Finally, d-amphetamine-evoked DA release in the SNc was found to have a significant, negatively correlated relationship with the phMRI motor cortex activation (r^2^ = 0.74, *P* = 0.027, Fig. 4E).

## 5 Discussion

Here, we used phMRI to assess the function of the cortico-basal ganglia circuit in a non-human primate model of dopamine deficiency to determine the possible relationships between phMRI signals and DA dysfunction. Our study supports a direct relationship between BOLD-responses and DA degeneration. First, BOLD-activation was highly correlated with a number of measures, including: 1) potassium- and d-amphetamine-evoked DA release in the putamen, 2) the number of surviving TH^+^ cells in the SNc, and 3) TH^+^ fiber density in the putamen. Moreover, TH^+^ fibers density was also correlated with phMRI responses in the putamen. These results are in line with those of a previous phMRI study in parkinsonian rhesus monkeys (Zhang et al., 2006)) in which phMRI responses to d-amphetamine were correlated with surviving DA neurons in the midbrain. The results from this and our previously published studies (Luan et al., 2008; Zhang et al., 2018; Zhang et al., 2006) support the concept that phMRI could be a useful tool for estimating the number of surviving DA neurons and terminals in the nigrostriatal pathway, in a non-invasive manner. This could provide pivotal information in assessing the disease state, the rate of progressive cell loss rate, and the effects of a disease modifying therapy over time.

BOLD-activation was also highly correlated with DA-related behavioral deficits. Because the same dose of APO was used for phMRI and behavioral testing, we believe that the APO-induced improvements in parkinsonism may have occurred in parallel to the normalization of the BOLD-signal in the motor cortex. (Hutchison et al., 1997; Stefani et al., 1999; Stefani et al., 1997). Along these lines, human deep brain stimulation (DBS) studies have shown that acute administration of APO causes a significant decrease in cell firing activity, which is paralleled by amelioration of PD hypokinesia as assessed by an intraoperatively-administered Unified Parkinson's Disease Rating Scale (UPDRS) examination. In a previously published study, hemiparkinsonian monkeys were treated with oral L-dopa (250 to 500 mg) twice a day for 5 days. We found that the treatment not only gradually and significantly reduced the initial L-dopa-induced rotational behavior (observed in the immediate days following L-dopa), but that the treatment also significantly reduced the amplitude of L-dopa-induced BOLD activations in the putamen (Chen et al., 1999). In a human phMRI study, significant correlations were demonstrated between the total motor UPDRS score and BOLD activation in the striatum, subthalamic nucleus, external segment of the globus pallidus and substantia nigra (Prodoehl et al., 2010).

Another novel observation from the current study is that the BOLD responses to APO administration were only observed in the putamen. The heterogeneous BOLD response to APO matched the pattern of heterogeneous changes of TH^+^ fibers in the putamen. This finding suggests that the BOLD responses to APO in the putamen could reflect the histopathological severity of degeneration of the DA system. In humans, postmortem analyses demonstrate heterogeneous changes in the putamen with the greatest reductions of dopamine uptake sites measured by [^3^H] mazindol binding located in the medial putamen (Porritt et al., 2005). In addition, [^18^F] dopa uptake detected by PET showed more severe loss of functional integrity of the nigrostriatal dopaminergic neurons in the posterior putamen in patients with PD. We believe that these findings could be attributed to the organization of the basal ganglia, which represents a complex extrapyramidal motor system, as opposed to the pyramidal motor system (corticobulbar and corticospinal pathways). The basal ganglia-thalamocortical circuits maintain somatotopic organization of movement-related neurons throughout the circuit mainly through the putamen while the caudate nucleus contributes to memory, learning, and cognitive functions, (for reviews, see Alexander et al., 1986; Herrero et al., 2002). Moreover, bradykinesia, which is a major clinical sign of PD, is more correlated with the putamen than the caudate nucleus or SN (Prodoehl et al., 2010).

In the current study, we selected late middle-aged animals because PD is an age-associated neurodegenerative disease that infrequently occurs before age 50 (Chu and Kordower, 2007; Ross et al., 2004; Silver, 2006) with most patients being older than 60 years at the time of diagnosis. Animals used for this study had ages ranging from 18 to 22 years old, which is the equivalent to ~54 to 66 years old in humans. These animals displayed stable parkinsonian features for over 3 years following the MPTP-treatments. A number of novel therapeutic strategies successfully tested in young parkinsonian monkeys have failed to reach their endpoint when tested clinically in patients with PD (Chu and Kordower, 2007). Thus, we hypothesize that this failure may be the result, at least in part, from failing to test these therapies in older animals, which may represent a much more clinically relevant model for translation.

Another focus of this study was to investigate how BOLD-activation in the motor cortex was associated with functional changes in other areas of the cortico-nigrostriatal loops. The results from the present study demonstrated that both dynamic BOLD-activations and basal glutamate levels were reduced in the primary motor cortex on the ipsilateral side to the MPTP lesion suggesting that stronger activations and higher glutamate levels were co-localized in the hemisphere contralateral to the MPTP administration. Few nonhuman primate studies have been conducted to directly compare these two parameters, particularly in parkinsonian monkeys. Previously, in vivo fMRI has been used to map the brain's hemodynamic responses to neuronal stimulations such as the susceptibility-based BOLD image contrast and the change in regional CBF (rCBF) (Silva and Koretsky, 2002; Silva et al., 1999; van Zijl et al., 1998). Results from those studies indicated that the effects of BOLD and rCBF appeared to reflect neuronal activity (Hoge et al., 1999; Logothetis et al., 2001; Ogawa et al., 2000; Silva and Koretsky, 2002), and were considered to be coupled tightly with neuronal activation via the metabolic demand of associated glutamate transportation (for a review, see Eulenburg and Gomeza, 2010). The results from the current study support the hypothesis that BOLD-activation is associated with basal glutamate levels. In a human study using a combined magnetic resonance spectroscopy and fMRI approach, Horn et al. (2010) found that glutamate and resting-state functional connectivity (judged by BOLD activation) were correlated in patients suffering with depression. Further support for a relationship between regional cortical connectivity and glutamate levels was reported by Duncan and colleagues (Duncan et al., 2011).

The results from our study in middle-aged rhesus monkeys with stable, long-term parkinsonism caused by MPTP administration confirm a close relationship between BOLD activation in the cortico-nigrostriatal loops induced by dopaminergic receptor stimulation and the severity of parkinsonism. In addition, BOLD activation was seen to correlate with 1) cortical levels of resting glutamate, 2) nigrostriatal dopamine overflow, and 3) histopathologically-determined numbers of dopaminergic neurons and terminals The results are in line with our previous studies that showed phMRI BOLD-responses induced by dopaminergic drugs can be used for assessing nigrostriatal dopamine function(Zhang et al., 2006), and for differentiating PD-related from age-related changes in basal ganglia function (Andersen et al., 2015). Future studies will need to be carried out to advance the development of this imaging strategy for application in humans.

## Author roles

1.Research project: A. Conception, B. Organization, C. Execution; 2. Statistical Analysis: A. Design, B. Execution, C. Review and Critique; 3. Manuscript Preparation: A. Writing of the first draft, B. Review and Critique.

JEQ:1B, 1C,2C, 3A

YA:1B, 1C, 3A

AHA:1A, 1B, 1C, 2B, 3B

PH:1A, 1B, 1C, 1B, 3B

RG:1B, 1C, 2C, 3B

ZG:3B

DMG:1A, 1B, 3B

GAG:1A, 1B, 3B

ZZ:1A, 1B, 1C, 2A, 2B, 3A

## Financial disclosures

JEQ: Employed by University of Kentucky, Consultant to Quanteon LLC

YA: Employed by University of Kentucky

AHA: Employed by University of Kentucky

PH: Employed by University of Kentucky

RG: Employed by University of Kentucky

ZG: Employed by University of Kentucky

DMG: Employed by University of Kentucky

GAG: Employed by University of Kentucky, Principal owner of Quanteon LLC

ZZ: Employed by University of Kentucky

## Funding

This study was supported by USPHS NIH grants NS39787 and NS50242.
